# 2-(4-Meth­oxy­benzyl­idene)-4,4-dimethyl-3,4-dihydro­naphthalen-1(2*H*)-one

**DOI:** 10.1107/S1600536810044387

**Published:** 2010-11-06

**Authors:** Mohamed Akhazzane, Hafid Zouihri, Jean-Claude Daran, Abdelali Kerbal, Ghali Al Houari

**Affiliations:** a Laboratoire de Chimie Organique, Faculté des Sciences Dhar el Mahraz, Université Sidi Mohammed Ben Abdellah, Fès, Morocco; b Laboratoire de Diffraction des Rayons X, Division UATRS, Centre National pour la Recherche Scientifique et Technique, Rabat, Morocco; c Laboratoire de Chimie de Coordination, 205 Route de Narbonne, 31077 Toulouse Cedex, France

## Abstract

The title compound C_20_H_20_O_2_, has the exocyclic C=C double bond in an *E* configuration. The two benzene rings form a dihedral angle of 72.92 (6)°.

## Related literature

For general background to dipolar-1,3 cyclo­addition reactions, see: Kerbal *et al.* (1988 ▶), Bennani *et al.* (2007 ▶); Al Houari *et al.* (2008 ▶). For a related structure, see: Al Houari *et al.* (2005 ▶).
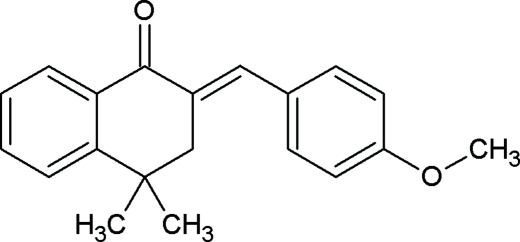


## Experimental

### 

#### Crystal data

C_20_H_20_O_2_
*M* *_r_* = 292.36
Monoclinic, 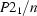

*a* = 11.8587 (3) Å
*b* = 8.7536 (2) Å
*c* = 14.9392 (4) Å
β = 96.527 (1)°
*V* = 1540.73 (7) Å^3^
*Z* = 4
Mo *K*α radiation
μ = 0.08 mm^−1^
*T* = 190 K
0.19 × 0.15 × 0.13 mm

#### Data collection

Bruker APEXII CCD detector diffractometer
15082 measured reflections
3159 independent reflections
2709 reflections with *I* > 2σ(*I*)
*R*_int_ = 0.029

#### Refinement

*R*[*F* ^2^ > 2σ(*F* ^2^)] = 0.039
*wR*(*F* ^2^) = 0.108
*S* = 1.08
3159 reflections
202 parameters
H-atom parameters constrained
Δρ_max_ = 0.21 e Å^−3^
Δρ_min_ = −0.22 e Å^−3^

### 

Data collection: *APEX2* (Bruker, 2005 ▶); cell refinement: *SAINT* (Bruker, 2005 ▶); data reduction: *SAINT*; program(s) used to solve structure: *SHELXS97* (Sheldrick, 2008 ▶); program(s) used to refine structure: *SHELXL97* (Sheldrick, 2008 ▶); molecular graphics: *PLATON* (Spek, 2009 ▶); software used to prepare material for publication: *publCIF* (Westrip, 2010 ▶).

## Supplementary Material

Crystal structure: contains datablocks I, global. DOI: 10.1107/S1600536810044387/ds2066sup1.cif

Structure factors: contains datablocks I. DOI: 10.1107/S1600536810044387/ds2066Isup2.hkl

Additional supplementary materials:  crystallographic information; 3D view; checkCIF report

## Figures and Tables

**Fig. 1.**
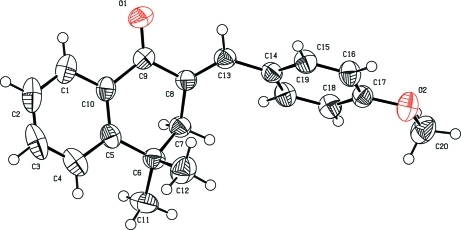
Two independent molecules of the title compound showing the atom-labelling scheme and 30% probability displacement ellipsoids.

**Fig. 2.**
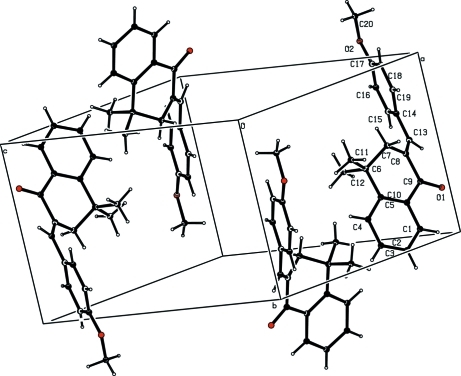
Partial packing view.

## supplementary crystallographic information

### Comment

Knowledge of the configuration and conformation of the title compound, (1), is necessary to understand its behaviour in dipolar-1,3 cycloaddition reactions (Bennani *et al.* 2007, Al Houari *et al.* 2008). To confirm the E configuration of the exocyclic C=C double bond, an X-ray crystal structure determination has been carried out.

In the title compound, C_20_H_20_O_2_, the two benzene rings form a dihedral angle of 72.92 (6)°.The cyclohexyl ring of the 3,4-dihydronaphthalen-1(2*H*)-one is distorted from a classical chair conformation, presumably due to conjugation of the planar annulated benzo ring (r.m.s. deviation 0.32 (13) A °). In the crystal, molecules are connected through C—H···O hydrogen bonds.

### Experimental

The synthesis of 2-(4-methoxybenzylidene)-4,4-dimethyl-3,4-dihydronaphthalen-1(2*H*)-one was achieved using the method reported by Kerbal *et al.* (1988), *i.e.* by a condensation of *para* anisaldehyde with 4,4-dimethyl-3,4-dihydronaphthalen-1(2*H*)-one in an alkaline medium in methanol.

### Refinement

All H atoms were fixed geometrically and treated as riding with C—H = 0.97 Å (methyne) and 0.93Å (aromatic) with *U*_iso_(H) = 1.2Ueq(C).

### Crystal data

**Table:**

C_20_H_20_O_2_	*F*(000) = 624
*M**_r_* = 292.36	*D*_x_ = 1.260 Mg m^−^^3^
Monoclinic, *P*2_1_/*n*	Mo *K*α radiation, λ = 0.71073 Å
Hall symbol: -P 2yn	Cell parameters from 1852 reflections
*a* = 11.8587 (3) Å	θ = 1.5–25.7°
*b* = 8.7536 (2) Å	µ = 0.08 mm^−^^1^
*c* = 14.9392 (4) Å	*T* = 190 K
β = 96.527 (1)°	Block, colourless
*V* = 1540.73 (7) Å^3^	0.19 × 0.15 × 0.13 mm
*Z* = 4	

### Data collection

**Table:**

Bruker APEXII CCD detector diffractometer	2709 reflections with *I* > 2σ(*I*)
Radiation source: fine-focus sealed tube	*R*_int_ = 0.029
graphite	θ_max_ = 26.4°, θ_min_ = 2.7°
ω and φ scans	*h* = −14→14
15082 measured reflections	*k* = −10→10
3159 independent reflections	*l* = −18→18

### Refinement

**Table:**

Refinement on *F*^2^	Primary atom site location: structure-invariant direct methods
Least-squares matrix: full	Secondary atom site location: difference Fourier map
*R*[*F*^2^ > 2σ(*F*^2^)] = 0.039	Hydrogen site location: inferred from neighbouring sites
*wR*(*F*^2^) = 0.108	H-atom parameters constrained
*S* = 1.08	*w* = 1/[σ^2^(*F*_o_^2^) + (0.0577*P*)^2^ + 0.2729*P*] where *P* = (*F*_o_^2^ + 2*F*_c_^2^)/3
3159 reflections	(Δ/σ)_max_ = 0.001
202 parameters	Δρ_max_ = 0.21 e Å^−^^3^
0 restraints	Δρ_min_ = −0.22 e Å^−^^3^

### Special details

**Table:**

Geometry. All e.s.d.'s (except the e.s.d. in the dihedral angle between two l.s. planes) are estimated using the full covariance matrix. The cell e.s.d.'s are taken into account individually in the estimation of e.s.d.'s in distances, angles and torsion angles; correlations between e.s.d.'s in cell parameters are only used when they are defined by crystal symmetry. An approximate (isotropic) treatment of cell e.s.d.'s is used for estimating e.s.d.'s involving l.s. planes.
Refinement. Refinement of *F*^2^ against ALL reflections. The weighted *R*-factor *wR* and goodness of fit *S* are based on *F*^2^, conventional *R*-factors *R* are based on *F*, with *F* set to zero for negative *F*^2^. The threshold expression of *F*^2^ > σ(*F*^2^) is used only for calculating *R*-factors(gt) *etc*. and is not relevant to the choice of reflections for refinement. *R*-factors based on *F*^2^ are statistically about twice as large as those based on *F*, and *R*- factors based on ALL data will be even larger.

### Fractional atomic coordinates and isotropic or equivalent isotropic displacement parameters (Å^2^)

**Table:**

	*x*	*y*	*z*	*U*_iso_*/*U*_eq_	
C6	0.04770 (10)	1.19124 (13)	0.42407 (7)	0.0258 (2)	
C10	0.25868 (10)	1.19809 (13)	0.41294 (7)	0.0269 (3)	
C9	0.25676 (9)	1.03468 (13)	0.37763 (7)	0.0270 (3)	
C5	0.16041 (10)	1.26897 (13)	0.43710 (7)	0.0265 (3)	
C14	−0.07348 (9)	0.83891 (13)	0.29360 (7)	0.0257 (2)	
C19	−0.04025 (10)	0.69666 (13)	0.32854 (8)	0.0291 (3)	
H19	0.0014	0.6914	0.3851	0.035*	
C15	−0.13830 (10)	0.84177 (13)	0.20924 (8)	0.0292 (3)	
H15	−0.1640	0.9350	0.1849	0.035*	
C8	0.15580 (9)	0.94675 (12)	0.40857 (8)	0.0268 (3)	
H8A	0.1489	0.8489	0.3779	0.032*	
H8B	0.1706	0.9271	0.4727	0.032*	
C16	−0.16482 (10)	0.70964 (13)	0.16147 (8)	0.0301 (3)	
H16	−0.2070	0.7146	0.1051	0.036*	
C7	0.04589 (9)	1.03173 (12)	0.39000 (7)	0.0246 (2)	
C18	−0.06740 (10)	0.56202 (13)	0.28164 (8)	0.0296 (3)	
H18	−0.0445	0.4683	0.3068	0.036*	
C17	−0.12881 (10)	0.56856 (12)	0.19710 (8)	0.0267 (3)	
C13	−0.04948 (9)	0.98441 (13)	0.34094 (7)	0.0264 (3)	
H13	−0.1092	1.0539	0.3362	0.032*	
C11	0.36517 (10)	0.94804 (15)	0.41279 (9)	0.0371 (3)	
H11A	0.4291	0.9941	0.3892	0.056*	
H11B	0.3585	0.8434	0.3937	0.056*	
H11C	0.3757	0.9524	0.4774	0.056*	
C4	0.16424 (12)	1.42072 (14)	0.46707 (8)	0.0349 (3)	
H4	0.0994	1.4656	0.4850	0.042*	
C1	0.35781 (11)	1.28557 (16)	0.41635 (9)	0.0373 (3)	
H1	0.4238	1.2414	0.4000	0.045*	
C3	0.26285 (13)	1.50403 (15)	0.47021 (9)	0.0423 (3)	
H3	0.2648	1.6048	0.4900	0.051*	
C20	−0.12557 (12)	0.29770 (14)	0.17537 (10)	0.0400 (3)	
H20A	−0.0442	0.2929	0.1850	0.060*	
H20B	−0.1533	0.2225	0.1315	0.060*	
H20C	−0.1561	0.2779	0.2311	0.060*	
C2	0.35928 (13)	1.43660 (16)	0.44363 (10)	0.0448 (4)	
H2	0.4256	1.4934	0.4441	0.054*	
C12	0.24585 (12)	1.04047 (16)	0.27413 (8)	0.0393 (3)	
H12A	0.1760	1.0898	0.2519	0.059*	
H12B	0.2463	0.9384	0.2507	0.059*	
H12C	0.3085	1.0968	0.2552	0.059*	
O2	−0.15974 (8)	0.44573 (9)	0.14344 (6)	0.0359 (2)	
O1	−0.03919 (7)	1.26029 (9)	0.43670 (6)	0.0340 (2)	

### Atomic displacement parameters (Å^2^)

**Table:**

	*U*^11^	*U*^22^	*U*^33^	*U*^12^	*U*^13^	*U*^23^
C6	0.0308 (6)	0.0244 (5)	0.0221 (5)	0.0023 (4)	0.0030 (4)	0.0019 (4)
C10	0.0296 (6)	0.0296 (6)	0.0204 (5)	−0.0040 (5)	−0.0016 (4)	0.0033 (4)
C9	0.0256 (6)	0.0295 (6)	0.0261 (6)	0.0004 (4)	0.0036 (4)	−0.0003 (4)
C5	0.0337 (6)	0.0247 (6)	0.0203 (5)	−0.0026 (5)	0.0005 (4)	0.0011 (4)
C14	0.0221 (5)	0.0262 (6)	0.0288 (6)	−0.0014 (4)	0.0036 (4)	−0.0002 (4)
C19	0.0300 (6)	0.0299 (6)	0.0264 (6)	−0.0019 (5)	−0.0012 (5)	0.0036 (5)
C15	0.0296 (6)	0.0245 (6)	0.0328 (6)	0.0021 (5)	0.0000 (5)	0.0042 (5)
C8	0.0263 (6)	0.0218 (5)	0.0319 (6)	0.0014 (4)	0.0021 (5)	0.0008 (4)
C16	0.0328 (6)	0.0306 (6)	0.0258 (6)	−0.0017 (5)	−0.0018 (5)	0.0018 (5)
C7	0.0256 (6)	0.0229 (5)	0.0255 (5)	0.0000 (4)	0.0042 (4)	0.0022 (4)
C18	0.0324 (6)	0.0229 (6)	0.0336 (6)	−0.0001 (4)	0.0036 (5)	0.0056 (5)
C17	0.0280 (6)	0.0247 (6)	0.0283 (6)	−0.0041 (4)	0.0071 (4)	−0.0014 (4)
C13	0.0257 (6)	0.0240 (6)	0.0297 (6)	0.0023 (4)	0.0041 (4)	0.0016 (4)
C11	0.0269 (6)	0.0397 (7)	0.0452 (7)	0.0031 (5)	0.0067 (5)	0.0022 (6)
C4	0.0503 (8)	0.0267 (6)	0.0265 (6)	−0.0013 (5)	−0.0014 (5)	−0.0019 (5)
C1	0.0312 (6)	0.0418 (7)	0.0374 (7)	−0.0077 (5)	−0.0030 (5)	0.0062 (6)
C3	0.0624 (9)	0.0276 (6)	0.0333 (7)	−0.0121 (6)	−0.0102 (6)	−0.0015 (5)
C20	0.0513 (8)	0.0238 (6)	0.0469 (8)	−0.0035 (5)	0.0143 (6)	−0.0020 (5)
C2	0.0457 (8)	0.0421 (8)	0.0423 (8)	−0.0204 (6)	−0.0140 (6)	0.0082 (6)
C12	0.0462 (8)	0.0442 (8)	0.0282 (6)	−0.0006 (6)	0.0074 (6)	−0.0039 (5)
O2	0.0481 (5)	0.0256 (4)	0.0338 (5)	−0.0052 (4)	0.0031 (4)	−0.0038 (3)
O1	0.0330 (5)	0.0291 (4)	0.0403 (5)	0.0058 (4)	0.0066 (4)	−0.0032 (4)

### Geometric parameters (Å, °)

**Table:**

C6—O1	1.2275 (13)	C7—C13	1.3414 (16)
C6—C7	1.4855 (15)	C18—C17	1.3855 (17)
C6—C5	1.4927 (16)	C18—H18	0.9300
C10—C1	1.3990 (17)	C17—O2	1.3661 (14)
C10—C5	1.4031 (16)	C13—H13	0.9300
C10—C9	1.5239 (16)	C11—H11A	0.9600
C9—C11	1.5330 (16)	C11—H11B	0.9600
C9—C12	1.5377 (16)	C11—H11C	0.9600
C9—C8	1.5381 (15)	C4—C3	1.3745 (19)
C5—C4	1.4009 (16)	C4—H4	0.9300
C14—C19	1.3898 (16)	C1—C2	1.383 (2)
C14—C15	1.3999 (16)	C1—H1	0.9300
C14—C13	1.4689 (15)	C3—C2	1.385 (2)
C19—C18	1.3901 (16)	C3—H3	0.9300
C19—H19	0.9300	C20—O2	1.4237 (15)
C15—C16	1.3766 (16)	C20—H20A	0.9600
C15—H15	0.9300	C20—H20B	0.9600
C8—C7	1.4990 (15)	C20—H20C	0.9600
C8—H8A	0.9700	C2—H2	0.9300
C8—H8B	0.9700	C12—H12A	0.9600
C16—C17	1.3925 (16)	C12—H12B	0.9600
C16—H16	0.9300	C12—H12C	0.9600
			
O1—C6—C7	122.40 (10)	C19—C18—H18	120.3
O1—C6—C5	120.69 (10)	O2—C17—C18	125.49 (10)
C7—C6—C5	116.80 (9)	O2—C17—C16	115.05 (10)
C1—C10—C5	117.88 (11)	C18—C17—C16	119.45 (10)
C1—C10—C9	120.38 (11)	C7—C13—C14	129.49 (10)
C5—C10—C9	121.60 (10)	C7—C13—H13	115.3
C10—C9—C11	111.52 (9)	C14—C13—H13	115.3
C10—C9—C12	108.26 (10)	C9—C11—H11A	109.5
C11—C9—C12	109.40 (10)	C9—C11—H11B	109.5
C10—C9—C8	110.32 (9)	H11A—C11—H11B	109.5
C11—C9—C8	107.49 (9)	C9—C11—H11C	109.5
C12—C9—C8	109.83 (10)	H11A—C11—H11C	109.5
C4—C5—C10	120.26 (11)	H11B—C11—H11C	109.5
C4—C5—C6	118.02 (11)	C3—C4—C5	120.70 (13)
C10—C5—C6	121.50 (10)	C3—C4—H4	119.7
C19—C14—C15	117.22 (10)	C5—C4—H4	119.7
C19—C14—C13	124.40 (10)	C2—C1—C10	121.09 (13)
C15—C14—C13	118.32 (10)	C2—C1—H1	119.5
C14—C19—C18	122.06 (10)	C10—C1—H1	119.5
C14—C19—H19	119.0	C4—C3—C2	119.43 (12)
C18—C19—H19	119.0	C4—C3—H3	120.3
C16—C15—C14	121.43 (10)	C2—C3—H3	120.3
C16—C15—H15	119.3	O2—C20—H20A	109.5
C14—C15—H15	119.3	O2—C20—H20B	109.5
C7—C8—C9	112.72 (9)	H20A—C20—H20B	109.5
C7—C8—H8A	109.0	O2—C20—H20C	109.5
C9—C8—H8A	109.0	H20A—C20—H20C	109.5
C7—C8—H8B	109.0	H20B—C20—H20C	109.5
C9—C8—H8B	109.0	C1—C2—C3	120.59 (12)
H8A—C8—H8B	107.8	C1—C2—H2	119.7
C15—C16—C17	120.32 (10)	C3—C2—H2	119.7
C15—C16—H16	119.8	C9—C12—H12A	109.5
C17—C16—H16	119.8	C9—C12—H12B	109.5
C13—C7—C6	117.12 (10)	H12A—C12—H12B	109.5
C13—C7—C8	127.53 (10)	C9—C12—H12C	109.5
C6—C7—C8	115.13 (9)	H12A—C12—H12C	109.5
C17—C18—C19	119.48 (10)	H12B—C12—H12C	109.5
C17—C18—H18	120.3	C17—O2—C20	118.20 (10)

### Hydrogen-bond geometry (Å, °)

**Table:**

*D*—H···*A*	*D*—H	H···*A*	*D*···*A*	*D*—H···*A*
C13—H13···O1	0.93	2.44	2.8034 (15)	104

## References

[bb1] Al Houari, G., Kerbal, A., Bennani, B., Baba, M. F., Daoudi, M. & Ben Hadda, T. (2008). *Arkivok*, pp. 42–50.

[bb2] Al Houari, G., Kerbal, A., El Bali, B. & Bolte, M. (2005). *Acta Cryst.* E**61**, o3330–o3331.

[bb3] Bennani, B., Kerbal, A., Daoudi, M., Filali Baba, B., Al Houari, G., Jalbout, A. F., Mimouni, M., Benazza, M., Demailly, G., Akkurt, M., Öztürk Yıldırım, S. & Ben Hadda, T. (2007). *Arkivok*, pp. 19–40.

[bb4] Bruker (2005). *APEX2* and *SAINT* Bruker AXS Inc., Madison, Wisconsin, USA.

[bb5] Kerbal, A., Tshiamala, K., Vebrel, J. & Laude, B. (1988). *Bull. Soc. Chim. Belg.* **97**, 149–161.

[bb6] Sheldrick, G. M. (2008). *Acta Cryst.* A**64**, 112–122. 10.1107/S0108767307043930 18156677

[bb7] Spek, A. L. (2009). *Acta Cryst.* D**65**, 148–155. 10.1107/S090744490804362X PMC2631630 19171970

[bb8] Westrip, S. P. (2010). *J. Appl. Cryst.* **43**, 920–925.

